# Progressive resistance training in cachectic head and neck cancer patients undergoing radiotherapy: a randomized controlled pilot feasibility trial

**DOI:** 10.1186/s13014-018-1157-0

**Published:** 2018-11-06

**Authors:** Manuel Grote, Cornelius Maihöfer, Martin Weigl, Patricia Davies-Knorr, Claus Belka

**Affiliations:** 1 Department of Radiation Oncology, University Hospital, LMU Munich, 81377 Munich, Germany; 2 Department of Orthopaedics, Physical Medicine and Rehabilitation, University Hospital, LMU Munich, 81377 Munich, Germany; 3 Present Address: Department of Health Promotion/Occupational Health Management, AOK Baden-Württemberg, 70191 Stuttgart, Germany; 4 Clinical Cooperation Group ‘Personalized Radiotherapy in Head and Neck Cancer’ Helmholtz Zentrum München, German Research Center for Environmental Health GmbH, 85764 Neuherberg, Germany

**Keywords:** Head and neck cancer, Cachexia, Resistance training, Radiotherapy, Randomized controlled trial, Feasibility study

## Abstract

**Background:**

Cancer cachexia is a prevalent symptom of head and neck neoplasms. The reduction in skeletal muscle mass is one of the main characteristics which can lead to poor physical functioning. The purposes of this pilot randomized controlled trial were to determine the feasibility of progressive resistance training in cachectic head and neck cancer patients during radiotherapy and to explore possible risks and benefits.

**Methods:**

Twenty cachectic participants with head and neck cancer receiving radiation were randomized to obtain either a machine supported progressive resistance training (*n* = 10) or usual care (n = 10). The training took place 3 times weekly for 30 min. Intervention included 3 exercises for major muscle groups with 8–12 repetition maximum for 3 sets each. Bioelectrical impedance analysis, hand-held dynamometry, Six-Minute Walk Test and standardized questionnaires for fatigue and quality of life were used for evaluating outcomes at baseline before radiotherapy (t1), after 7 weeks of radiotherapy (t2) and 8 weeks after the end of radiotherapy (t3).

**Results:**

All participants (*n* = 20) completed the trial. No serious adverse events occurred. At the initial assessment the cachectic patients had already lost 7.1 ± 5.2% of their body weight. General fatigue (score 10.7 ± 3.3) and reduced quality of life (score 71.3 ± 20.6) were prevalent in cachectic head and neck cancer patients even before radiotherapy. An average improvement of weight loading for leg press (+ 19.0%), chest press (+ 29.8%) and latissimus pull-down (+ 22.8%) was possible in the intervention group. Participants had at least 13 training sessions. The outcome measures showed nonsignificant changes at t2 and t3, but a trend for a better course of general fatigue and quality of life at t2 in the intervention group.

**Conclusions:**

Despite advanced tumor stage and burdensome treatment the intervention adherence is excellent. Progressive resistance training in cachectic head and neck cancer patients during radiotherapy seems to be safe and feasible and may have beneficial effects of general fatigue and quality of life.

**Trial registration:**

ClinicalTrials.gov, NCT03524755. Registered 15 May 2018 - Retrospectively registered.

## Background

Head and neck cancer (HNC) is the ninth most common cause of cancer death [1]. In 2012 an estimated 529,000 new cases of cancer of the oral cavity and pharynx occurred worldwide. In Germany data describe incidence estimates of 13,800 and relative 5-year-survival rates between 48% for men and 61% for women in 2010 [2].

Tobacco use and alcohol consumption are the most significant risk factors for the development of oral cavity neoplasms [3]. A strong multiplicative effect for alcohol interacting with smoking further increases risk [4]. In addition human papillomavirus infection has a potential role mainly in the increased incidence for oropharyngeal cancers [5].

HNC and its medical treatments can lead to a complex interacting range of adverse effects which includes fatigue [6], altered body image [7], modified nourishment [8], psychosocial distress [9], shoulder dysfunction [10], trismus [11], dysphagia [12], xerostomia [13], mucositis [14], cachexia [15, 16], all resulting in reduced quality of life [17].

Modern radiotherapy techniques can reduce acute and chronic side effects. Intensity modulation [18, 19] and image guided [20] radiotherapy allow improved dose distribution thus sparing critical organs and improving quality of life [21, 22].

Considering the above mentioned debilitating symptoms, rehabilitation for HNC patients is warranted and potentially beneficial after medical treatment. In recent years it has been shown that physical exercises after and even during treatment relieve many of the side effects experienced by patients with various neoplasms [23–25].

Cancer cachexia and its key feature of weight loss do not develop to the same extent in every cancer entity. One of the high-risk populations developing cachexia is HNC patients [26, 27]. A generalized inflammatory state with high interleukin-6 levels is seen as a critical factor for muscle wasting [28, 29]. Loss of muscle mass leads to asthenia which is responsible for poor physical functioning [30].

The primary objectives of this pilot feasibility randomized controlled trial (RCT) were to determine the practicability of recruitment and the feasibility of progressive resistance training (PRT) during radiotherapy (RT) for cachectic HNC patients. Secondary objectives were to gather preliminary results for safety and responsiveness of the training.

## Methods

### Trial design

This study is a pilot, 2-arm, RCT. Between June 2013 and January 2015 a total of 20 patients at the university hospital Munich, Großhadern were randomly assigned to receive PRT (intervention group) or usual care (control group) during RT. Ethical clearance was obtained from institutional ethics committee (Project-Nr. 531–12) and written informed consent obtained from all participants before initiating study activities. Participants with a cancer diagnosis of the lung, pancreas, esophagus, head and neck, colon, rectum or anus were subsequently reviewed for inclusion and exclusion criteria. Study inclusion criteria were: (1) planned inpatient or outpatient RT, (2) ≥ 18 years of age, (3) diagnosed state of cachexia (weight loss greater than 5% over the past 6 months) or pre-cachexia (unintentional weight loss of 5% or less of usual body weight during the last 6 months). Patients with (1) metastatic disease, (2) severe neurological problems or other contraindications for the PRT were excluded. The project coordinator screened potential participants for eligibility via MOSAIQ®-Software (Radiation Oncology Information System). This software manages the aspects of the radiation oncology program and collects all information of the patients and makes them accessible. Study information sheets were sent to patients in the form of invitation letters. Participants who met the diagnosed tumor localization were met at the time of computer tomography-planning to explain the study, screen again for eligibility, and obtain informed consent. Registered participants were planned for baseline assessment. Participants were allocated at random to the control or exercise group via blocked randomization in sealed envelopes.

Baseline data for all participants were ascertained via medical records and patient interview. This included demographic information, UICC-status (Union internationale contre le cancer, tumor staging system), comorbidities and the results of blood samples. One study coordinator completed all assessments to enhance patient compliance. Body weight loss percentage was calculated via the individuals’ body weight 6 months before (in retrospect) and the current body weight. Participants completed two questionnaires: The Multidimensional Fatigue Inventory (MFI) [31] which consists of 20 items that measure subgroups (general, physical and mental fatigue as well as reduced motivation and reduced activity) of fatigue with a 5-point Likert scale and the Functional Assessment of Anorexia/Cachexia Therapy (FAACT) questionnaire [32, 33] which registers well-being for physical, social/family, emotional and functional aspects of quality of life and additional concerns in cachexia with 5-point Likert scale for 39 items. To measure physical performance the Six-Minute Walk Test (6MWT) [34] was applied. This assessment instrument includes walking distance as well as heart rate, pulse oximetry (Contec Medical Systems CO., Ltd., Model: CMS50E), fatigue (with a rating of perceived exertion (RPE) from 0 to 10) and dyspnea (RPE 0–10) before and after the test. To document changes in muscle force, strength of the functional muscle group for elbow flexion in supine position as well as of knee extension in sitting position (in each case right and left) was tested via hand-held dynamometry (Mecmesin Ltd., Broadbridge Heath, West Sussex, RH12 3IR, UK) for isometric maximal muscle strength [35]. Bioelectrical impedance analysis (BIA; AKERN SRL, BIA 101 New Edition) was executed to assess the adaption in body composition [36]. Patients were lying supine on a therapy table. The measurements took place at the right side of the body with the tetrapolar-technique of 4 standard electrodes on the surface of the hand and the foot. Constant frequency of 50 kHz and 400 μA constant current in electrical resistance of the body were used to measure resistance and reactance. The above mentioned assessments were carried out at baseline (t1), after 7 weeks of radiotherapy (t2) and 8 weeks after radiotherapy (t3).

Before each training session the current condition of the patient was accurately gathered. This included communication with the responsible nurse and/or physician about the ongoing state, including the results of the latest investigations such as blood pressure, temperature and laboratory investigations. As a last safety action the patient was asked if he felt able to carry out the training. Heart rate during training was monitored by a chest strap that sends the current heart rate by a radio signal to a wristwatch. It was payed attention that the participant’s heart rate never exceeded the maximum heart rate measured during the 6MWT and that the heart rate became slower in the breaks during the sets and the changes to another exercise machine. After the training each inpatient was personally carried back to the ward and asked for any complaints. Furthermore, the responsible nurse and/or physician were informed about the patients return to the ward and any noticeable situations during the training.

The exercise intervention was undertaken in the hospitals department of physical and rehabilitation medicine and based on standardized but individualized training protocols. All exercises were carried out within the individuals given limb range of motion and in a dynamic way without defined execution time for the concentric and eccentric phase. Goal of the very first training was to determine the training load set for hypertrophic adaption. Because of the vulnerable patient group a submaximal step by step approach within 3 sets and 8–12 repetitions was chosen. The first two sets acted as both muscular and movement adaption. The last set served as submaximal one-repetition maximum estimation. The evaluation of the training prescription was done in real-time by a supervising physiotherapist.

The training protocol consisted of a warm up period for 5 min on a bicycle ergometer or an upper body cycle with individual selectable wattage. A leg press, a latissimus pull-down and a chest press (Kaphingst) formed the three equipment supported core exercises. All exercises were performed with 8–12 repetitions and 3 sets. After each set and during the changes to another exercise machine a break of maximum 60 s was assured. After each machine the patients rated their perceived exertion from 0 to 10. The weight loading was increased at the next workout if RPE < 7. For the two upper limbs exercise a progression of 2.5 kg weight loading and for the lower limbs exercise a progression of 5.0 kg weight loading was implemented. The exercises were supervised all the time for performance and safety reasons by physiotherapists experienced in oncology rehabilitation and certified in medical training therapy.

For exercise participants with concomitant chemotherapy to their RT protocol timing for the training intervention was organized e.g. between the changes of 24 h constant-rate infusion. In the rare cases where this was not possible the exercise intervention was adjusted in order to protect the peripheral venous catheter in the Vena mediana cubiti by limiting an exercise in the range of motion or by replacing the two upper body machine supported exercises by: low pulley seated bench cable crossover and low pulley lateral extensions because of the almost static elbow angulation.

The control group received usual care. This could include inpatient physiotherapy if prescribed by the ward physician. Muscle strengthening techniques were not part of the usual care.

### Statistical analyses

Descriptive statistics were calculated to check for data normality. Normality was tested using Kolmogorov-Smirnov test. Single variables were compared using Student’s t-test and dichotomous outcomes were analyzed using a Chi-squared test or Fisher’s exact test as appropriate. Between group analysis was done using independent t-test or Mann-Whitney test. Statistical significance was set as *p* < .05. The statistic package IBM SPSS 23 was used for these analyses.

#### Sample size

Physical outcomes have been tested following exercise interventions in cancer studies. The outcome of physical function was analyzed with meta-analytic procedures and the overall weighted mean effect size for two-group comparisons was 0.52 [37]. This effect size calculated with an α-error probability of 0.05 and a power of 0.8 for a two-sided t-test resulted in a required sample size of 120 participants. For the control and intervention group 60 participants each.

## Results

On review of the recruitment process in July 2014 it was noted that there were only HNC patients included (one patient with esophageal cancer). Afterwards solely HNC patients were screened for inclusion criteria to generate a homogenous analysis.

### Patient recruitment (Fig. 1)

Between June 2013 and January 2015 100 eligible cancer patients scheduled for RT treatment were subsequently screened. 20 patients were included giving a recruitment rate of 20%. 80 patients were excluded for following reasons: metastases (*n* = 20), second cancer diagnosis and/or relapse (*n* = 18), no exercise therapy possible due to other comorbidities (*n* = 8), no interest (*n* = 16), no time (*n* = 4), RT at non-study site (*n* = 3) and other reasons (*n* = 11). All included patients were randomized into intervention and control groups before beginning RT. The completion rate was 100%.

**Fig. 1:**
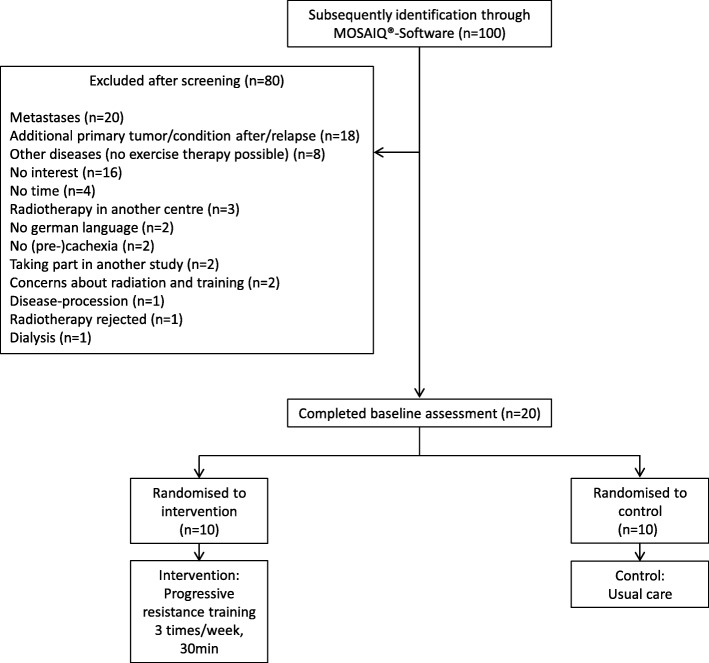
Participant flow chart

### Feasibility outcomes

The 10 participants from the training group achieved a total of 168 training sessions, with a mean of 16.8 training sessions (range 13–25) during the course of RT. A total of 84 h of training could be accomplished with 30 min for each session. This is a mean of 8.4 h of training for each exercising patient. A mean of 32 days of RT (range 25–33) results in 2.7 training sessions of the planned 3 sessions per week (range 1.7–3.8). Mean time between hospital admission and the first training session was 1 working day (range 0–3). Mean time between the last training session and the last RT was 3 working days (range 13–0). Explanations for not completing all planned training sessions included pain due to severe mucositis (4 patients). Change from in- to outpatient status during RT led to reduced training time slots because participants had to organize the time for training considering RT time (4 patients). Three participants reported paused progression in the exercise of a latissimus pull-down due to abdominal tension following recently placed percutaneous endoscopic gastrostomy (PEG).

None of the participants reported muscle strains to the physiotherapist. No infections of peripheral venous catheter, no chemotherapy extravasation and no other adverse events during or after the training were observed.

Inpatient physiotherapy was prescribed in 3 participants of the control group. Chest physiotherapy to facilitate expectoration and movement therapy for the shoulder girdle after neck dissection were contents of the therapy sessions. A total of 17 therapy sessions with 15–35 min were carried out.

### Clinical characteristics

The study population (*n* = 20) consists of upper aerodigestive tract neoplasms of which 85% (*n* = 9 intervention, *n* = 8 control) are head and neck squamous cell carcinoma (HNSCC). Whereas the remaining 15% are patients with tumors of the esophagus (*n* = 1 intervention) or the salivary gland (n = 2 control).

Table 1 shows the baseline characteristics of the patients. Both of the groups were comparable at baseline with regard to age, sex, cancer stage, alcohol and smoking in the medical history, medical treatment, carried out neck dissection and PEG. Of the 20 patients 15 were men and 11 had a history of either smoking and/or alcohol abuse. 35% received RT as single medical treatment. All patients received fractionated radiotherapy with 60–70 Gy (five fractions/week). Thirteen patients received concurrent chemotherapy, most of them cisplatin 20 mg/m^2^ and 5-fluoruracil 600 mg/m^2^ body surface area on day 1–5 and repeated as of day 29. Neck dissection and percutaneous endoscopic gastrostomy has been performed in 11 patients each.

**Table 1:** Clinical characteristics

Variable	All (*n* = 20)	Intervention (*n* = 10)	Control (*n* = 10)	*P* value
Age (years)	60.9 ± 11.3 (27–82)	60.2 ± 4.7 (50–66)	61.5 ± 15.7 (27–82)	0.807
Sex				0.303^b^
Men	15 (75%)	9 (90%)	6 (60%)	
Women	5 (25%)	1 (10%)	4 (40%)	
Cancer stage^a^				0.420
Stadium I/II	6 (30%)	3 (30%)	3 (30%)	
Stadium III/IV (M0)	14 (70%)	7 (70%)	7 (70%)	
Alcohol abuse, yes	5 (25%)	3 (30%)	2 (20%)	1.000^b^
Smoking, yes	6 (30%)	3 (30%)	3 (30%)	1.000^b^
Chemoradiation, yes	13 (65%)	8 (80%)	5 (50%)	0.350
Neck dissection, yes	11 (55%)	5 (50%)	6 (60%)	1.000
Feeding tube, yes	11 (55%)	7 (70%)	4 (40%)	0.370^b^

^a^*UICC* Classification

^b^Fisher’s exact test

The comparison of baseline values for blood sample data (C-reactive protein *p* = 0.922, hemoglobin *p* = 0.710 and interleukin-6 *p* = 0.620) and bodyweight (*p* = 0.094) or weight loss (*p* = 0.274) was similar between both groups. At the time of giving consent to the study patients had lost at an average 7.1 ± 5.2% of their body weight (72.1 ± 12.7 kg) as compared to 6 months before. C-reactive protein (standard value ≤0.5 mg/dl) was only slightly increased (1.0 ± 1.5 mg/dl) and hemoglobin (standard value 13.5–17.5 g/dl) was at the lower limit (13.4 ± 1.3 g/dl). Elevated interleukin-6 levels (12.0 ± 18.2 pg/ml, standard value ≤5.9 pg/ml, *n* = 18) were also shown.

All participants (*n* = 20) were assessed for functional capacity with the 6MWT. It shows a mean of 475 ± 101 m (intervention group 500 ± 77 m, control group 450 ± 119 m, between group analyses *p* = 0.284). Hand-held dynamometry for testing maximal isometric strength demonstrated for knee extension of right lower extremity 30.2 ± 11.9 kp, left lower extremity 30.0 ± 11.9 kp (between group comparison *p* = 0.254 respectively *p* = 0.077). Isometric testing of the upper extremity via flexion of the elbow showed for the right side a mean of 18.8 ± 6.0 kp and left 19.0 ± 5.8 kp (between group comparison *p* = 0.171 respectively *p* = 0.433).

The results of the questionnaires for fatigue (Multidimensional Fatigue Inventory) and quality of life in anorexia/cachexia (Functional Assessment of Anorexia/Cachexia Therapy) are presented in Table 2. These show that both groups were balanced at baseline. Fatigue subscale scores range from 8.0–11.5 and the FACT-G score is 77.9 ± 15.2, demonstrating a moderate symptom occurrence before medical treatment.

**Table 2:** Baseline data for fatigue and quality of life in anorexia/cachexia

Variable	All (*n* = 20)	Intervention (*n* = 10)	Control (*n* = 10)	*P* value
Fatigue				
MFI general fatigue (4–20)	10.7 ± 3.3 (5–16)	11.3 ± 3.7 (5–16)	10.1 ± 2.9 (6–14)	0.393
MFI physical fatigue (4–20)	11.0 ± 4.1 (4–20)	12.0 ± 5.0 (4–20)	10.0 ± 2.9 (6–14)	0.393
MFI reduced activity (4–20)	11.5 ± 4.0 (4–20)	11.5 ± 5.0 (4–20)	11.4 ± 2.7 (6–15)	0.912
MFI reduced motivation (4–20)	8.8 ± 3.9 (4–16)	8.6 ± 4.2 (4–16)	9.0 ± 3.9 (5–16)	0.853
MFI mental fatigue (4–20)	8.0 ± 4.1 (4–20)	7.6 ± 4.9 (4–20)	8.3 ± 3.4 (4–13)	0.436
Quality of life in anorexia/cachexia				
FACT-G total score (0–108)	77.9 ± 15.2^a^ (49–104)	80.1 ± 11.2^b^ (61–96)	75.7 ± 18.8^b^ (49–104)	0.730

^a^n = 18, ^b^n = 9 (three patients had a overall item response rate smaller than 80%)

We found comparable baseline values for muscle (*p* = 0.917) and fat (*p* = 0.279) mass between the groups. A mean of 41.8 ± 7.7% (29–59) muscle mass and 19.9 ± 8.3% (3–35) fat mass was shown (*n* = 20)

### Training effects (Table 3)

The increase in general (*p* = 0.879), physical (*p* = 0.478) and mental (*p* = 0.696) fatigue is not significantly different between t1 and t3 comparing intervention and control group. Similarly, the reduced activity increased in both groups (control 8%, intervention 11%) to a non-significantly extent (*p* = 0.700). In the reduced motivation dimension, the intervention group showed a decrease of 7%, while the control group indicated an increase of 14% between t1 and t3 (*p* = 0.437).

**Table 3:** Training effects of fatigue dimensions, quality of life and fat and lean mass at test intervals t1, t2 and t3

Variable	Intervention *n* = 10	Control *n* = 10	*P* Value (t1-t3)
Fatigue (4–20)			
General Fatigue pre	11.3 ± 3.7	10.1 ± 2.9	0.879
General Fatigue post	12.3 ± 5.3	14.1 ± 3.5	
General Fatigue follow-up	11.8 ± 4.3	11.9 ± 4.6	
Physical Fatigue pre	12.0 ± 5.0	10.0 ± 2.9	0.478
Physical Fatigue post	13.5 ± 4.5	13.8 ± 2.8	
Physical Fatigue follow-up	13.3 ± 5.0	11.8 ± 5.1	
Mental Fatigue pre	7.6 ± 4,9	8.3 ± 3.4	0.696
Mental Fatigue post	9.6 ± 4.4	9.7 ± 4.0	
Mental Fatigue follow-up	8.3 ± 2.3	9.2 ± 2.3	
Reduced Activity pre	11.5 ± 5.0	11.4 ± 2.7	0.700
Reduced Activity post	13.1 ± 4.8	14.8 ± 2.8	
Reduced Activity follow-up	12.4 ± 4.4	12.6 ± 4.2	
Reduced Motivation pre	8.6 ± 4.2	9.0 ± 3.9	0.437
Reduced Motivation post	9.2 ± 4.1	9.9 ± 2.2	
Reduced Motivation follow-up	8.0 ± 2.5	10.3 ± 4.8	
Quality of life in anorexia/cachexia (0–104)			
Quality of Life pre	80.1 ± 11.2	75.7 ± 18.8	0.891
Quality of Life post	56.5 ± 18.4	53.4 ± 19.8	
Quality of Life follow-up	64.4 ± 18,4	59.5 ± 26.5	
Bioelectrical Impedance analysis (kg)			
Fat mass pre	14.7 ± 8.6	16.4 ± 7.0	0.545
Fat mass post	12.1 ± 7.0	12.9 ± 6.2	
Fat mass follow-up	10.2 ± 6.6	13.2 ± 5.3	
Lean mass pre	58.6 ± 4.9	54.4 ± 12.5	0.267
Lean mass post	57.8 ± 8.9	53.5 ± 11.9	
Lean mass follow-up	59.1 ± 7.1	52.7 ± 12.1	

Variables: *Fatigue* Multidimensional Fatigue Inventory (possible scoring 4–20), *Quality of life in anorexia/cachexia* Functional Assessment of Anorexia/Cachexia Therapy *FAACT* questionnaire (possible scoring 0–104), *Bioelectrical Impedance analysis* Body composition measurement (in kilogram); pre (t1): before radiotherapy, post (t2): after 7 weeks of radiotherapy and follow-up (t3): 8 weeks after radiotherapy represent the time at which the outcome was assessed. Analysis of variance for repeated measurements with the two factors Time and Group, *p* < 0.05

Comparing the decrease in quality of life in the control and intervention group is non-significant (*p* = 0.891). The relative decrease between t1 and t3 was 7% in the intervention group and 19% in the control group. In the period between t1 and t2 the quality of life of the control group decreased by 27% and in the intervention group by 18%.

The intervention group shows a 30% loss of fat mass, the control group decreased 20% of fat mass in the observation period. In the t2-t3 interval, the untreated group increased its fat mass by 2% and the intervention group decreased 16%. While the lean mass in the intervention group increased by 1% during the observation period, it decreased by 3% in the control group (*p* = 0.267).

### Training progression

**Fig. 2:**
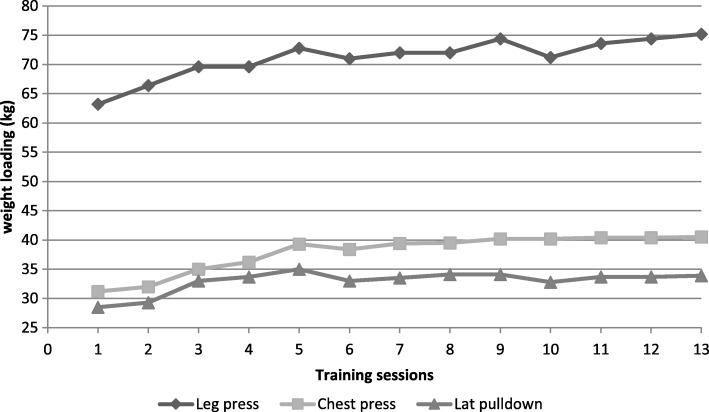
Weight loading progression during the first 13 training sessions for the equipment supported exercises

All of the 10 participants had a minimum of 13 training sessions. Figure 2 displays the mean course of the training regarding weight loading (in kg) for the 3 equipment supported exercises: leg press, chest press and latissimus pull-down in the first 13 sessions. Increases in strength can be shown for each of the equipment supported exercises. Especially during the first 5 sessions of the training it was possible to increase the load substantially (see Fig. 2). Afterwards the progression in loading tended to level off. During the whole course of 13 training sessions there was an average increase of 19.0% of weight loading for the leg press; starting from 63.2 kg in the first session and 75.2 kg in the last. Weight loading for the chest press began at 31.2 kg to 40.5 kg at last; equivalent to an improvement of 29.8%. Maximum weight loading for the latissimus pull-down was in the fifth training session (starting with 28.5 kg and ending 35.0 kg; 22.8% weight loading progression).

## Discussion

The primary intention of this prospective trial was to determine the feasibility of individually tailored PRT in cachectic patients diagnosed with HNC. The main finding of the present study is that even in cachectic patients with HNC receiving RT a PRT seems to be safe and well tolerated. None of the participants reported muscle strains. No infections of the peripheral venous catheter, chemotherapy extravasation or other adverse events were reported during or after the training. Additionally, it was possible to increase the training loads within the first 5 sessions and maintain them throughout the medical treatment.

When considering physical strength as an outcome parameter it is important to be able to quantify muscle strength. Strength measurement in this trail needed to be easy to apply, mobile and inexpensive. A hand-held dynamometric device was therefore used to measure maximal isometric muscle strength. This pilot study was able to show 294 ± 117.7 Newton for knee extension at baseline. Although Knols et al. [38] found similar data (278 ± 93.6 Newton) their sample was more than 10 years younger (49.45 ± 14.85 vs. 60.9 ± 11.3) and in various types and stages of cancer chemotherapy treatment. The comparison of strength values measured via a hand-held dynamometry are not generalizable as can be seen by the fact that reliability is significantly influenced by the strength of the tester [39]. Therefore, the measurements should always be carried out by the same tester within a study (as it was done in our trial). The 6MWT is a valid measurement for physical performance in cancer patients. This was also demonstrated in this study with a mean distance of 475 ± 101 m achieved. When compared to the walking distance (652 m) in healthy population based samples [40] the discrepancy could be explained as a result of the decreased physical function caused by cachexia. Cachexia not only reduces physical activity which leads to noteworthy changes in performance status. It also directly impacts on quality of life which is a complex outcome measure in cancer patient trials. Both cachectic NSCLC and HNC patients seem to have a similar reduction in quality of life related to anorexia/cachexia. A FAACT score of 111 ± 17 was reported [41] for 26 patients with anorexia-cachexia syndrome in an outpatient cancer care situation. Among our patients a FAACT sore of 112 ± 23 seems to be comparable. This is surprising considering that HNC patients regularly have to cope with tracheostoma or feeding tubes that are required to bypass tumor-related complications. This often leads to changes in eating habits which also can have an effect to the adaption and change of muscles when they are used. Especially resistance training provokes an increase in the cross-sectional area of the muscle fiber types, referred to as muscle hypertrophy, which is able to gain muscle weight if factors such as age, sex, nutrition and training status have a positive basis in an individual. Strength gains during the early phases of resistance training are a common adaption of the neuro-muscular system [34, 35]. It could be shown that during the first five training sessions patients were able to progressively increase loading compared to the following sessions. Consequently the neuro-muscular-system was able to process more loading in this early phase of training. The training resistance could also be increased more easily at the beginning because no side effects such as painful mucositis had yet occurred. The performance stagnation in the further course of the training could be explained by the fear of pushing oneself additionally or to the begin of adverse side effects to the medical therapy.

BIA serves to determine the body composition. Percentage body fat was measured with BIA in our trial at baseline. Sun et al..... [42] measured BIA in 591 healthy volunteers with a mean age of 42.15 ± 10.27 (19–60) and found a body fat mass of 32.85 ± 8.00 (10.6–58.3) percent. In Isenring et al’s research HNC patients were randomized before RT in a nutrition intervention (NI) group or a usual care (UC) group [43]. Both groups in Isenring et al’s research were comparable at baseline with respect to fat mass in kg (19.4 ± 4.8 vs 20.5 ± 9.7; *p* = 0.640). A calculated mean of 25.1% fat mass in the NI group and 26.7% in the UC group is comparable to our analyzed fat mass of 19.9 ± 8.3%. The lower fat mass in this study could be explained with the cachectic state of our patients. Research into body composition status with respect to skeletal muscle mass in advanced esophageal cancer patients undertaken by Ida et al. 30 patients underwent BIA before neoadjuvant chemotherapy [44]. They found a mean muscle mass of 24.9 ± 0.8 kg with a mean body weight of 59.1 ± 1.7 kg which corresponds to 42.1% muscle mass proportion. With 37% Takekiyo et al. [45] found approximately equal values for muscle mass at baseline. In their analysis eighty-six patients underwent BIA before hematopoietic stem cell transplantation. When compared to our results of 41.8 ± 7.7% muscle mass in an already cachectic patient sample seems to be quite a lot. Cancer cachexia is however a heterogeneous phenomenon which can vary through the time spectrum (pre-cachexia, cachexia, and refractory cachexia), tumor type, site and mass. Metabolic change as well as reduced food intake, co-morbidity, pre-existing sarcopenia, cancer therapy and genetic predisposition can play a role in cancer cachexia [46].

### Training effects

Although the results of the MFI did not show any significant differences between groups over the study period, it should be noted that in 4 out of 5 subcategories, the control group had a higher average percentage increase in fatigue scores over the interval t1 to t2. The main difference was found in the dimension general fatigue. In this dimension the fatigue score in the intervention group worsened by 9%, whereas the control group experienced a 40% worsening. This is in line with a Cochrane review that found a significant beneficial effect of tumor related fatigue in patients who performed aerobic exercise and positive, although nonsignificant, trends for resistance training and other forms of exercise therapy [47].

In the t1-t2 period the questionnaire FAACT showed a notable result for the quality of life of cachectic tumor patients. Here it is indicated that the decrease in quality of life by 18%, compared to the control group with 27%, the intervention group had less loss of quality of life over the period of medical therapy. Cancer patients often have many mental and physical side effects as a result of their cancer or treatment. Some studies have suggested that physical exercises may be helpful in reducing negative outcomes and improving the quality of life of cancer patients during their treatment [48].

The via bioelectrical impedance analysis measured lean mass essentially represents the mass of the muscles, the organs, the skeletal system and the central nervous system. The lean mass is calculated on the basis of body water (total body water / 0.73 = lean mass). A relatively constant hydrogenation of 73% is assumed. This could also basically mean that the courses of the lean mass and the total body water are similar. The control group loses 3% (intervention + 1%) lean mass over the entire study period. However, as the subjects are tumor patients who suffer from oral fluid and food intake limitations, the results are arguable. Fat mass is calculated from the weight difference of lean mass and body weight. Both groups lost a noteworthy proportion of their fat mass throughout the study period. By contrast, the decrease in muscle mass only affected the period from t1 to t2. There, both groups equally relieved muscle mass. During the same period, the intervention group lost 18% and the control group 21% of their fat mass. This result is consistent with the statement that in tumor patients fat is degraded to a greater degree than muscle mass [49]. This result allows important conclusions: Firstly, a nutritional intervention with a fatty component should be incorporated as soon as possible and in the long term in order to reduce the early and preferential breakdown of fat mass. On the other hand, the musculature necessary for the functioning of patients in their daily environment appears to be a non-primary source of supply for the maintenance of cachexia.

In our sample of cachectic HNC patients the selected assessments for the measurement of physical performance, quality of life and body composition could be implemented without difficulty.

The present pilot study has certain limitations. The small sample size and non-blinding of assessors and therapists are major limitations which reflect lack of time and budget in pilot studies. Especially in physical activity studies selection bias is ubiquitous and generates an inevitable risk for data extrapolation. Appropriately powered analyses were not sought due to the pilot character of this study. However, randomization, the presence of a control group and an approach to characterize cachectic body components as an outcome measure for resistance training are major strengths of this study.

## Conclusion

In this pilot RCT a new therapy focus on the high-risk patient group of cachectic HNC patients during RT has been established. Progressive resistance training during RT for cachectic HNC patients is feasible. In our sample of patients, it was well tolerated and safe, but due to the small sample size we may have missed less frequent adverse events. In the early phase of training patients could progressively manage more resistance. This suggests that cachectic HNC patients might benefit from PRT in the early phase of RT. No significant benefits in fatigue and QOL were found, but the slightly better outcomes for general fatigue and QOL in the intervention group and the feasibility results can be used to design a future definitive clinical trial.

## Abbreviations

6MWT: Six-minute walk test
BIA: Bioelectrical impedance analysis
FAACT: Functional assessment of anorexia/cachexia therapy
HNC: Head and neck cancer
HNSCC: Head and neck squamous cell carcinoma
MFI: Multidimensional fatigue inventory
NSCLC: Non-small cell lung cancer
PEG: Percutaneous endoscopic gastrostomy
PRT: Progressive resistance training
RPE: Rating of perceived exertion
RT: Radiotherapy
